# Clinical Benefits of Combining Different Visualization Modalities in Neurosurgery

**DOI:** 10.3389/fsurg.2019.00056

**Published:** 2019-10-01

**Authors:** Karl-Michael Schebesch, Katharina Rosengarth, Alexander Brawanski, Martin Proescholdt, Christina Wendl, Julius Höhne, Christian Ott, Hans Lamecker, Christian Doenitz

**Affiliations:** ^1^Department of Neurosurgery, University Medical Center Regensburg, Regensburg, Germany; ^2^Department of Radiology, University Medical Center Regensburg, Regensburg, Germany; ^3^1000shapes GmbH, Berlin, Germany

**Keywords:** fluorescence-guided surgery, fluorescein sodium, YELLOW 560 nm, KINEVO, tumor segmentation, confocal endomicroscopy, CONVIVO, brain tumors

## Abstract

The prevailing philosophy in oncologic neurosurgery, has shifted from maximally invasive resection to the preservation of neurologic function. The foundation of safe surgery is the multifaceted visualization of the target region and the surrounding eloquent tissue. Recent advancements in pre-operative and intraoperative visualization modalities have changed the face of modern neurosurgery. Metabolic and functional data can be integrated into intraoperative guidance software, and fluorescent dyes under dedicated filters can potentially visualize patterns of blood flow and better define tumor borders or isolated tumor foci. High definition endoscopes enable the depiction of tiny vessels and tumor extension to the ventricles or skull base. Fluorescein sodium-based confocal endomicroscopy, which is under scientific evaluation, may further enhance the neurosurgical armamentarium. We aim to present our institutional workup of combining different neuroimaging modalities for surgical neuro-oncological procedures. This institutional algorithm (IA) was the basis of the recent publication by Haj et al. describing outcome and survival data of consecutive patients with high grade glioma (HGG) before and after the introduction of our Neuro-Oncology Center.

## Introduction

Modern oncological neurosurgery is marked by the consensus that all surgical interventions should aim to attain complete tumor resection without affecting neurological function. This dogma was finally agreed upon because low tumor burden and good neurological function has been repeatedly shown to form the basis of any successful adjuvant treatment modality and to result in prolonged progression-free and overall survival, whilst preserving good quality of life (1–3).

The combination of different imaging modalities for pre-operative planning and intraoperative guidance should always aim at clearly identifying the targeted tumor that can be extremely heterogeneous. Currently, the integration of various functional imaging data, such as functional magnetic resonance imaging (fMRI), into neuronavigation allows for increased safety when approaching vulnerable, eloquent structures. Fluorescence-guidance can further support the neurosurgeon's eye and experience in visualizing the tumor mass, scattered tumor spots, infiltrated zones and rims, and patterns of blood flow, and can ultimately confirm a tumor-free cavity (4, 5).

However, in the daily routine of a neuro-oncology center (NOC), it is always mandatory to obtain and employ the desired imaging modality in a cost-effective, fast, and uncomplicated manner. Patients must be protected from redundant assessments, and, above all, “technical overkill” in the operating theater must be avoided. Furthermore, critical self-reflection and monocentric or multicenter complication analyses should be consistently generated and published to properly outline advantages and disadvantages, as well as clinical benefits and limitations of the institutional algorithm (IA). This approach is the only way that the most important factors, namely the surgical skills and experiences of neurosurgeons, are effectively supported by innovative technical adjuncts.

In 2017, we published our outcome and survival data of consecutive patients with high grade glioma since the introduction of a certified NOC in 2009. Since 2009, all patients with HGG at our department have been treated according to our IA. The current investigation has shown clear benefits in neurological outcome, progression-free survival, and overall survival in comparison to the equivalent data collected before 2009 (6).

The impact of the NOC organization in terms of improving survival in patients with glioblastoma has been described previously (6), but the detailed description of the workflow and institutional algorithm have yet to be reported. Therefore, the aim of this paper is to present our approach of combining different visualization modalities that are pre- and post-operatively employed not solely for the intraoperative depiction of the targeted area, but also for advanced pre-operative unmasking of the tumor structure, invasiveness, environment, and adherences. This combination enables sophisticated planning of the surgical approach including positioning, craniotomy, and dissection. We strongly believe that the interaction of a visual armamentarium provides neurosurgeons with more sensitivity and purposefulness, especially in complex neuro-oncologic procedures.

## Results

When a new patient with a suspected brain tumor is referred to our center, structural MRI is usually initially completed (see Table 1). Tumors without Gadolinium enhancement are further analyzed with FET-PET, which influences the choice of intraoperative fluorescent dyes. Proximity of the tumor to eloquent areas results in a functional workup (fMRI and Diffusion Tensor Imaging, DTI). All imaging data are then pre-processed and segmented to provide the essential information of each imaging modality. The final therapeutic decision is made after 3D-visualization and demonstration of the case, followed by stereotactic biopsy, open biopsy/partial resection, or gross tumor removal. The choice of fluorescent dye depends on the level of gadolinium enhancement and the FET-PET result (see Table 2).

**Table 1:** Overview of our institutional imaging workup for common intracranial lesions: magnetic resonance imaging (MRI) protocol for patients with intracranial tumors include T1 spin echo and T2 turbo spin echo sequences with 1 mm and isotropic 3D sequences (1 mm) including T1 w/gadolinium contrast (3D multiplane reformation, MPR), T2 (3D sampling perfection with application optimized contrast using different flip angle evolution, SPACE), 3D fluid attenuation inversion recovery, FLAIR, and constructive interference in steady-state (CISS MRI).

**Suspected diagnosis**	**Low grade glioma**	**High grade glioma/metastasis**	**Vascular lesions (aneurysms/AVM)**	**Skull base tumor**
MRI	SE T1 w/o CM, SE T2, 3D MPR w/contrast, 3D T2, 3D FLAIR	SE T1 w/o CM, T2, 3D MPR w/contrast, 3D T2, 3D FLAIR	SE T1 w/o CM, SE T2, 3D MPR w/contrast, 3D TOF, TWIST	SE T1 w/o CM, SE T2, 3D MPR w/contrast, 3D TOF, T2^*^ CISS
CT thin-sliced skull base (1 mm)	^*^✓		✓	✓
FET-PET	✓	^*^✓		
fMRI, rsfMRI	✓	✓	^*^✓	
DTI	✓	✓	^*^✓	
CFD simulation			^*^✓	

✓ mandatory,

*✓ *optional*.

*3D time-of-flight (TOF) and time-resolved angiography with interleaved stochastic trajectories (TWIST) sequences are obtained in vascularized tumors. Positron emission tomography (PET) with O-(2-[^18^F]fluoroethyl)-L-tyrosine ([^18^F]FET) (FET-PET) is used for non-contrast enhancing and recurrent contrast-enhancing gliomas. Functional MRI (fMRI) and resting state (rs) fMRI are conducted for visualizing functional areas*.

**Table 2:**
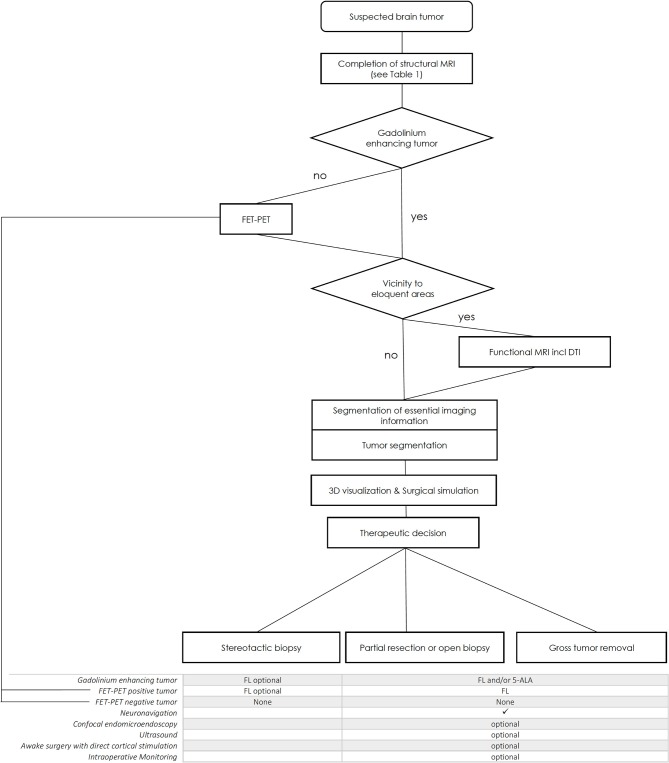
Institutional algorithm (IA).

The following features of the IA (Table 2) were selected because of their fundamental scientific interest:

### Pre-operative Workup–Completion of Structural and Metabolic Neuroimaging

When a new patient with a suspicious cranial lesion is referred to our center, neuroimaging is quickly completed according to our institutional imaging requirements (see Table 1) that include magnetic resonance imaging plus isotropic 3D sequences, computed tomography (CT), or 18F-fluorethyl tyrosine (FET)-/18F-fluordesoxyglucose (FDG)-positron emission tomography (PET). According to the recommendations of our daily case conference, patients are evaluated neuro-psychologically and consecutively undergo functional MRI and diffusion tensor imaging (DTI).

In 2018, we published a small series of patients with glioma that had not shown any contrast enhancement in the MRI, yet presented with distinct metabolic activity in the FET-PET (7). These patients had been suitable for fluorescence-guided surgery with Fluorescein Sodium (FL) under the YELLOW 560 nm filter (Carl Zeiss Meditec, Oberkochen, Germany). A possible correlation between FET-PET active tumors and fluorescent staining was assumed, facilitating surgical performance. Our results supported the data by Rapp et al. and Pirotte et al. who had outlined the diagnostic value of FET-PET for diagnosing hot spots inside gliomas. Clearly, FET-PET increases intraoperative diagnostic accuracy and helps to establish the most exact histopathological diagnosis (8, 9).

### Pre-operative Workup–fMRI and DTI

**Figure 1:**
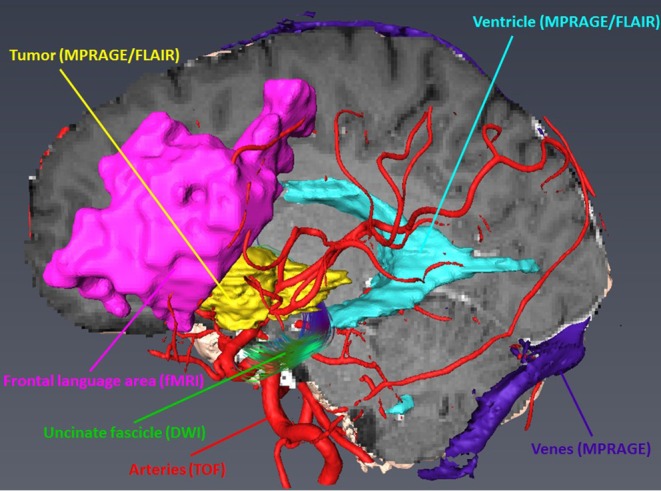
Integration and three-dimensional display of different structural and functional modalities in AMRIA. The original imaging modality used for visualization is annotated in brackets.

The aim of pre-surgical functional imaging is to maximize the resection of lesions (tumors or metastases) as well as to minimize post-operative functional deficits. Both factors increase post-operative health-related quality of life (10) and are prognostic factors for successive interventions involving radiation- and/or chemotherapy (11, 12). In our center, pre-surgical functional imaging is conducted with fMRI and resting state functional (rsf) MRI for the functional localization of cortical gray matter; diffusion-weighted imaging is used to determine subcortical white matter tracts. fMRI is exclusively carried out with 3-Tesla Siemens MRI scanners. Protocols for mapping the sensorimotor homunculus, language location and lateralization, and memory or retinotopic organization of the visual cortex depend on the localization of the lesion. Non-compliant or severely disabled patients may undergo rsfMRI that is also used in the case of patients with language barriers or in very young patients, awake patients with their eyes open, or in narcotized patients. fMRI data are analyzed with (S)tatistical(P)arametric(M)apping12 (www.fil.ion.ucl.ac.uk/spm/software/spm12/) including the LI-toolbox (13) and rsfMRI data with in-house generated Matlab scripts including parts of DPARSF and the GIFT-toolbox (http://mialab.mrn.org/software/gift/). According to fiber tracking, diffusion-weighted images (DWI) are acquired by means of 3T and 1.5T MRI scanners, either with 30 directions and 3 mm isotropic resolution or 64 directions with 2 mm isotropic resolution depending on the disease and patient characteristics. The following fiber tracking modeling can be done either deterministically with AMIRA (FEI Visualization Sciences, France) or probabilistically by using a modified FSL pipeline [employing Bayesian Estimation of Diffusion Parameters Obtained using Sampling Technique (BEDPOSTX)]. Fiber tracking is conducted in advance, when mainly pyramidal tracts, arcuate fascicles, uncinated fascicles, and optic tracts are shown. All functional results (fMRI, rsfMRI, and DTI) can be integrated into AMIRA (FEI Visualization Sciences, France) (see Figure 1).

### Pre-operative Workup–Tumor Segmentation

**Figure 2:**
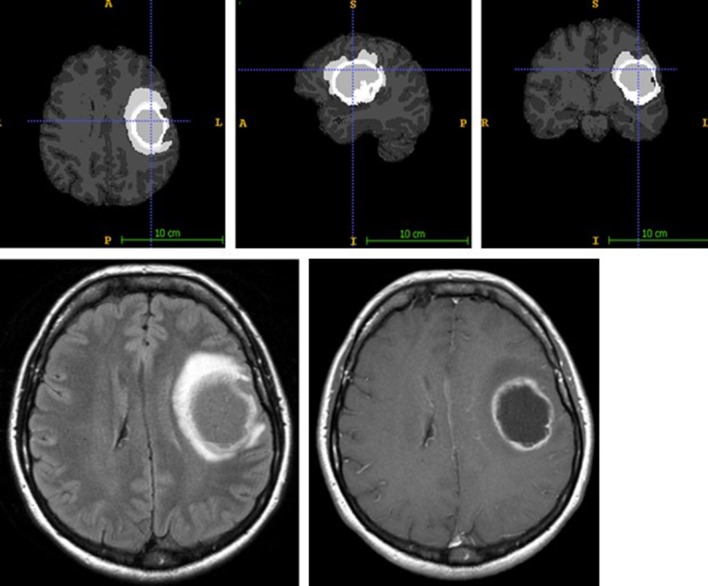
ANTsR tumor segmentation result of glioblastoma, showing labels for contrast-enhancing tumor (very light gray), necrosis (dark gray), edema (light gray) (upper row; contrast non-enhancing tumor label is not depicted), and the corresponding FLAIR (lower row left) and T1 with contrast agency (lower row right) MR image.

Automatic pre-operative brain tumor segmentation is a powerful tool for surgical planning that allows the extraction of tumor characteristics (contrast-enhancing and non-contrast-enhancing compartments, edema, and necrosis) from surrounding healthy brain tissue by means of a set of MR modalities (T1 with and without contrast agent, T2, and FLAIR) (14). Such information may be very useful for guiding surgical interventions by defining the extent of resection or the area of resection. There are a number of segmentation and tumor detection algorithms, ranging from classification or clustering approaches to deep learning algorithms (15, 16). In our department, we have established a tumor segmentation pipeline based on ANSTsR [advanced normalization tools (ANTs) and the R statistical project] using random forest-derived probabilities to determine different tumor tissues (17) (see Figure 2).

### Pre-operative Workup−3D Visualization and Surgical Simulation

Pre-operatively, a special simulation software is employed that condenses the essential information of the different imaging modalities in a 3D-viewer. This software enables the surgeon to pre-operatively envision the operative field as realistically as possible, simulating elements such as patient and head positioning in a virtual clamp, craniotomy, and corticotomy in a virtual OR setting. This software has been developed and modified by our department according to the specific needs of neurosurgeons.

Every pre-operative imaging modality provides unique information to characterize the lesion and its surroundings. For the sake of visual clarity, it is essential to condense and organize the extensive amount of anatomical and functional data. For this reason, we developed an advanced visualization software tool called NeuroVis, aimed at improving the understanding of anatomical and functional relations for pre-operative planning and intraoperative guidance in a virtual reality setting.

**Figure 3:**
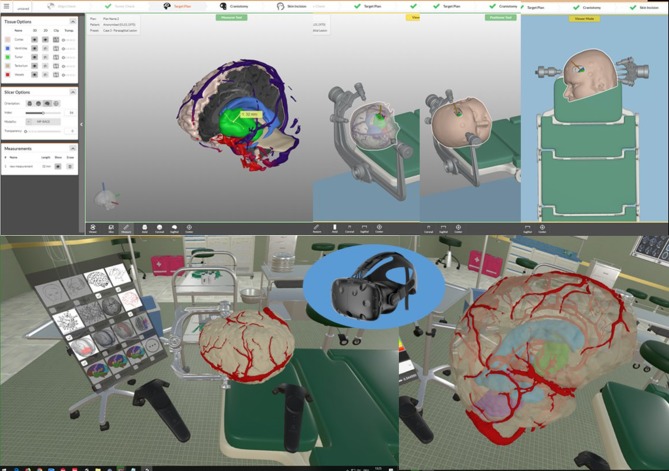
Pre-operative visualization with NeuroVis 3D (upper row) and in a virtual reality setting (lower row) using a head-mounted display.

The essential information of every image modality is segmented and co-registered with the 3D MPR image stack by means of a semi-automated workflow with AMIRA. Segmented data are then visualized with our browser- based rendering 3D viewer NeuroVis (developed at our institute in collaboration with 1000shapes GmbH, Berlin). This tool enables neurosurgeons to plan individually tailored treatment strategies. Planning steps include skin incision, craniotomy, and intraoperative positioning of the patient and the head clamp in a realistic operative setting. We have also established an interface to a virtual reality setting using UNITY (Unity Tec., USA) and a head-mounted display (HTC, Taiwan). These tools help to pre-operatively envision the operative field as realistically as possible (see Figure 3). The prepared plan can also be transferred to a navigation system using the stl format for intraoperative use.

### Intraoperative Workup–FET-PET Positive Non-enhancing Tumors and Fluorescence

The question of how to achieve the most effective tumor reduction without affecting relevant motor, language, and visual tracts continues to be highly controversial, particularly in non-contrast enhancing gliomas in eloquent regions of the brain. Besides the integration of functional data into neuro-navigation and conducting surgery in an awake setting, fluorescent visualization of the tumor may significantly increase the quality of resection and speed up surgery. Our recently published experience in patients with non-enhancing gliomas with distinct metabolic activity in FET-PET and clear intraoperative fluorescent staining (7), in addition to the report by Bowden et al. (18), supports the potential supplementary role of FL in cases of low or intermediate grade glioma with high metabolic activity.

### Intraoperative Workup–Fluorescein-Enhanced Confocal Endomicroscopy

Because of its unspecific accumulation in areas of disrupted blood-brain barrier (BBB), FL can be applied widely irrespective of the tumor histology in HGG (19–22), cerebral metastases (23, 24), lymphomas (25, 26), meningiomas (27), neuromas (28), and brain abscesses (29) for guiding microsurgical resection under the dedicated light filter. Furthermore, FL-based confocal endomicroscopy can be employed *in vivo* to obtain multiple digital biopsies to visualize the tissue texture, enabling distinct histological evaluation by a (remote) neuropathologist (4, 30–33). This approach circumvents having to wait for the frozen section, enabling the surgeon to rapidly identify the histological origin of the lesion and delineate the tumor border much more precisely. However, since this technique is still under scientific evaluation, clinical data are required to outline the potential significance of this approach (30).

### Decision Making

This evaluation and the information provided by our neuropsychologist determine whether surgery is planned in an awake setting or under sedation. Neuro-navigation integrating all neuroimaging devices (fMRI, CT, and PET) is clinically routine.

Prior to surgery, we decide on the intraoperative visualization tools to be used, depending on the suspected etiology of the tumor, the anatomical localization, the expansion into eloquent areas and fiber tracts, and subject to the surgical approach. With the exception of intraoperative magnetic resonance imaging (iMRI), our neurosurgical department comprises all conventional technical adjuncts and personal competences of a tertiary academic neurosurgical center: (a) Fluorescence with dedicated light filters: fluorescein sodium (FL, YELLOW 560 nm filter), 5-aminolevulinic acid (5-ALA, BLUE 400 nm filter), and indocyanine green (ICG, FLOW 800 module); (b) Neuronavigation, ultrasound; (c) Intraoperative monitoring (IOM); (d) Endoscopy; and (e) Confocal endomicroscopy (still under scientific evaluation).

### Illustrative Cases

#### Case #1

**Figure 4:**
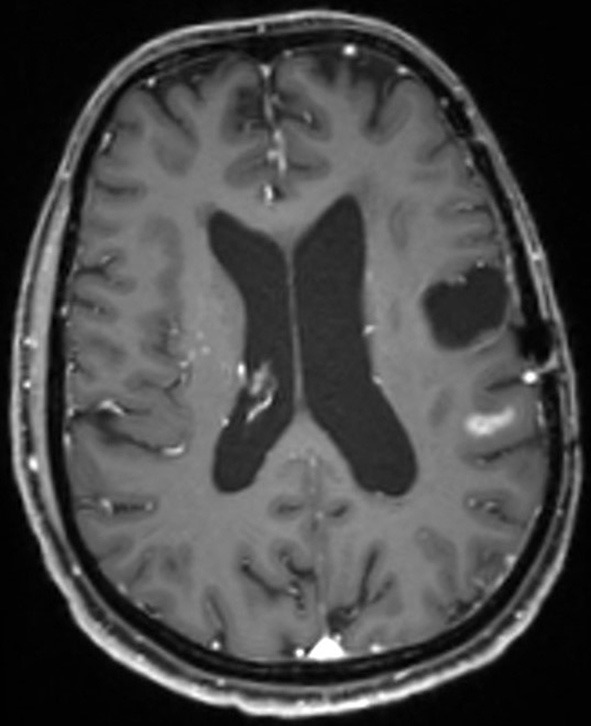
Pre-operative MR image (T1-weighted sequence, axial plane) showing new contrast-enhancement distant to the former resection cavity.

**Figure 5:**
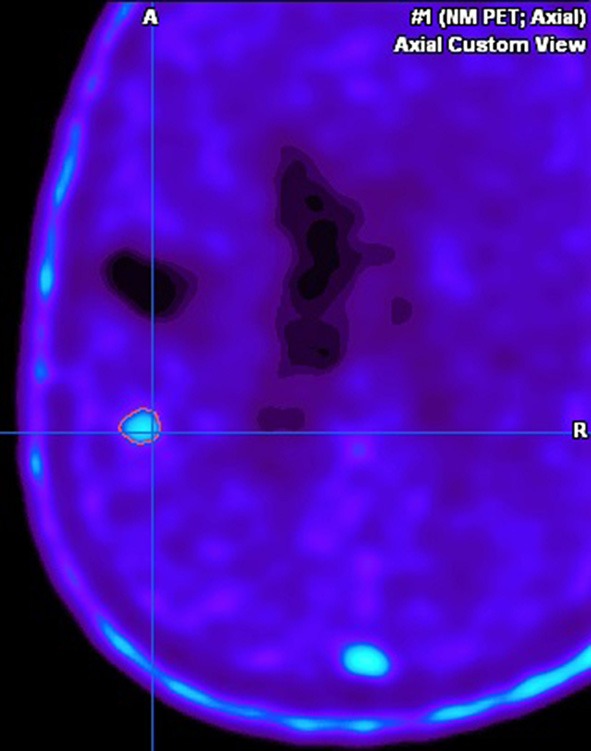
Pre-operative FET-PET image, displayed in the navigational software, showing strong metabolic activity in the suspicious area.

**Figure 6:**
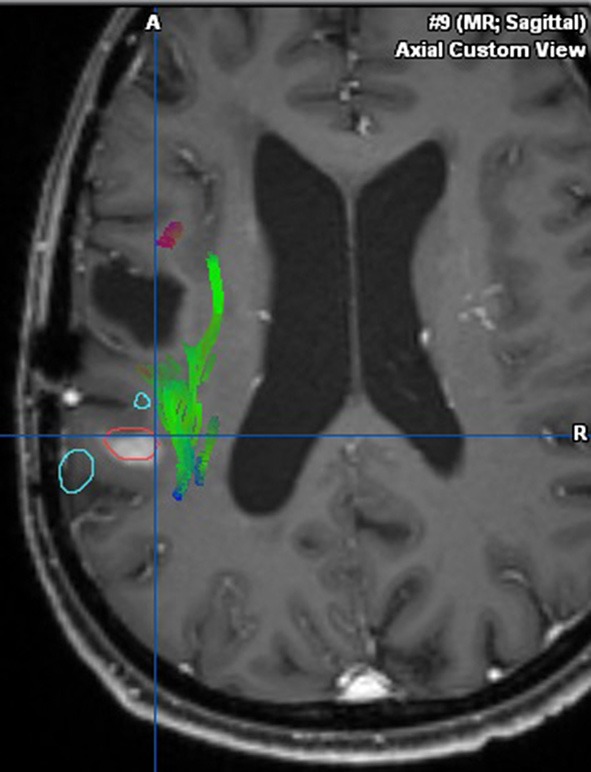
Integration of fMRI, DTI, and FET-PET (in red) into the navigational software, unmasking the proximity to eloquent area (arcuate fasciculus in green, language-associated activation in cyan).

**Figure 7:**
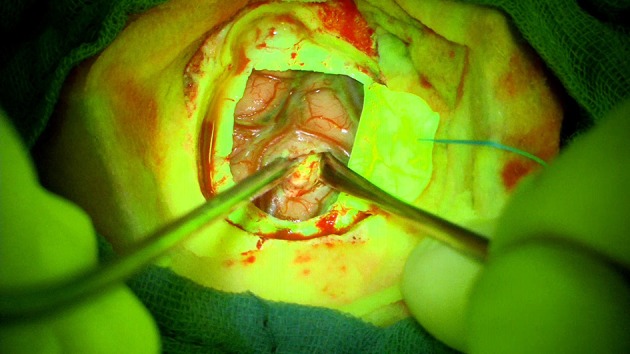
Strong fluorescent staining of the tumor under the YELLOW 560 filter.

**Figure 8:**
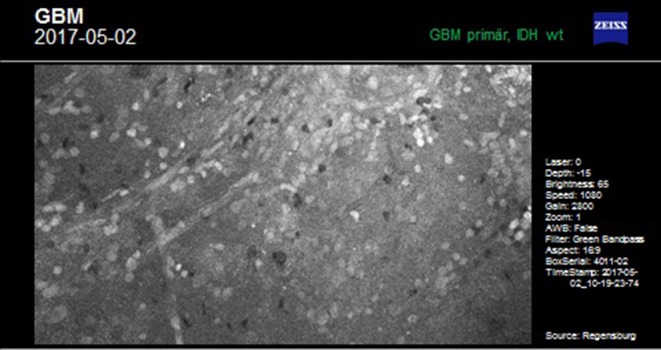
*Ex vivo* examination of tumorous tissue with the confocal endomicroscope.

This case describes our approach to the treatment of a remotely recurrent, eloquently located, contrast-enhancing astrocytoma WHO III of a 51-year-old female patient. The pre-operative neuroimage-workup consisted of conventional MRI (T1-weighted, axial sequence, Figure 4), FET-PET (displayed in navigational software, axial sequence, Figure 5), and neuro-navigation with integrated fMRI, DTI, and FET-PET (Figure 6). The patient was surgically treated in an awake-awake setting under fluorescence-guidance with FL (5 mg/kg) and the YELLOW 560 nm filter (Figure 7), and the removed tumor was examined *ex vivo* with the confocal endomicroscope (Figure 8). Early post-operative contrast-enhanced MRI showed complete removal of the contrast-enhanced lesion that was histologically confirmed as astrocytoma WHO III.

This case was selected because the pre-operative neuroimage-workup distinctively supported the indication for surgery. The FET-PET result ruled out pseudo-progression or post-radiation necrosis because of the detected strong metabolic activity that could be matched to the suspicious contrast enhancement in the pre-operative MRI. The functional area was displayed by means of fMRI and DTI, clearly showing proximity but no infiltration to language-associated activation. Finally, fluorescence-guided technique with FL impressively visualized the tumor *in vivo* and *ex vivo*. Taken together, the included imaging modalities resulted in complete removal of the tumor without any functional deterioration.

#### Case #2

**Figure 9:**
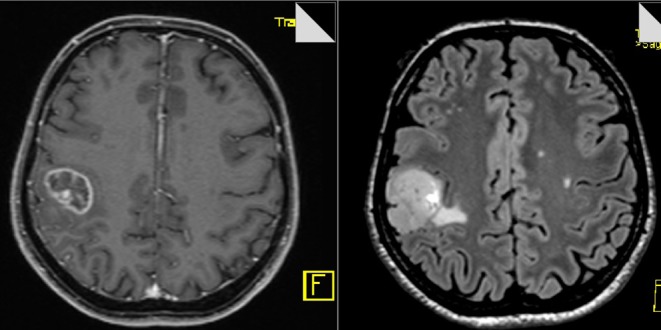
Pre-operative MR image (T1-weighted and FLAIR sequences, axial planes) showing circular contrast-enhancement and moderate edema.

**Figure 10:**
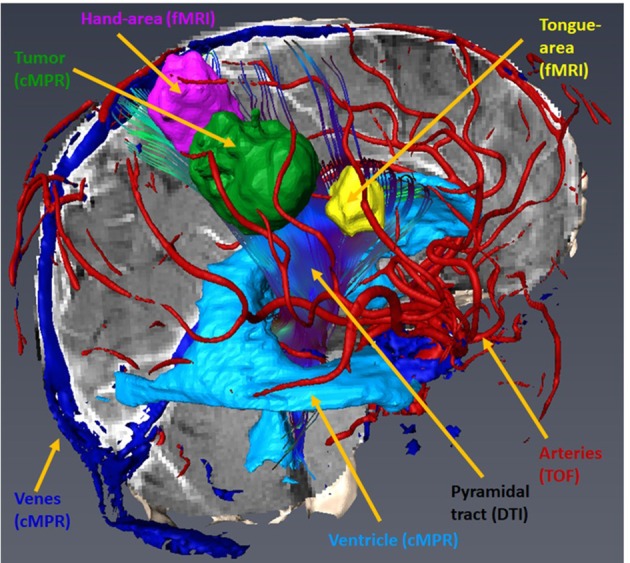
Sagittal view of the Amira 3D-reconstruction of the cortex, the vessels, the ventricle, the tumor (green), the hand motor associations areas (magenta), the tongue motor association area (yellow), and the pyramidal tract.

**Figure 11:**
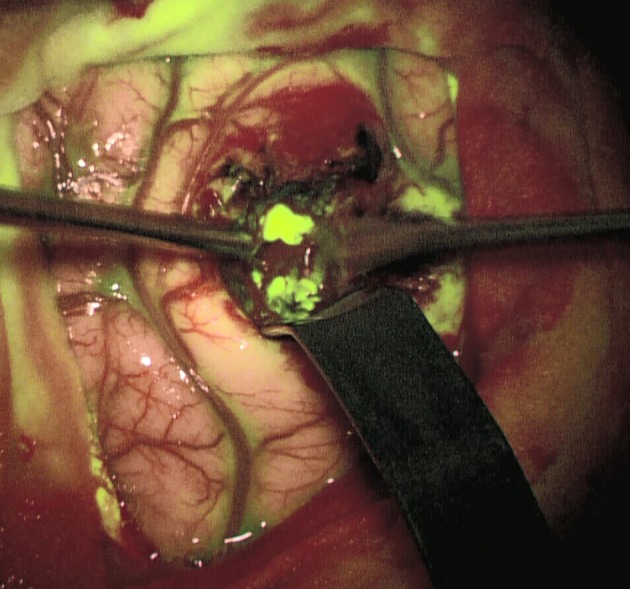
Strong fluorescent staining of the tumor under the YELLOW 560 filter.

**Figure 12:**
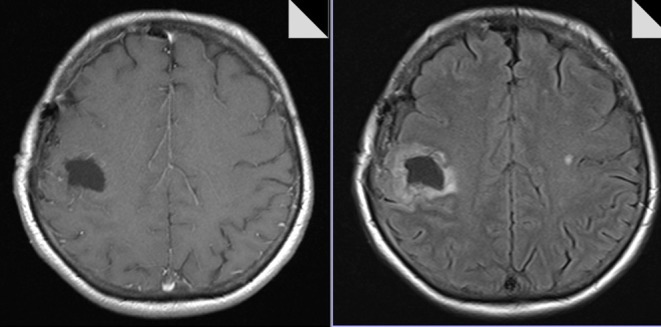
Post-operative MR image (T1-weighted and FLAIR sequences, axial planes) confirming complete removal of the tumor.

**Figure 13:**
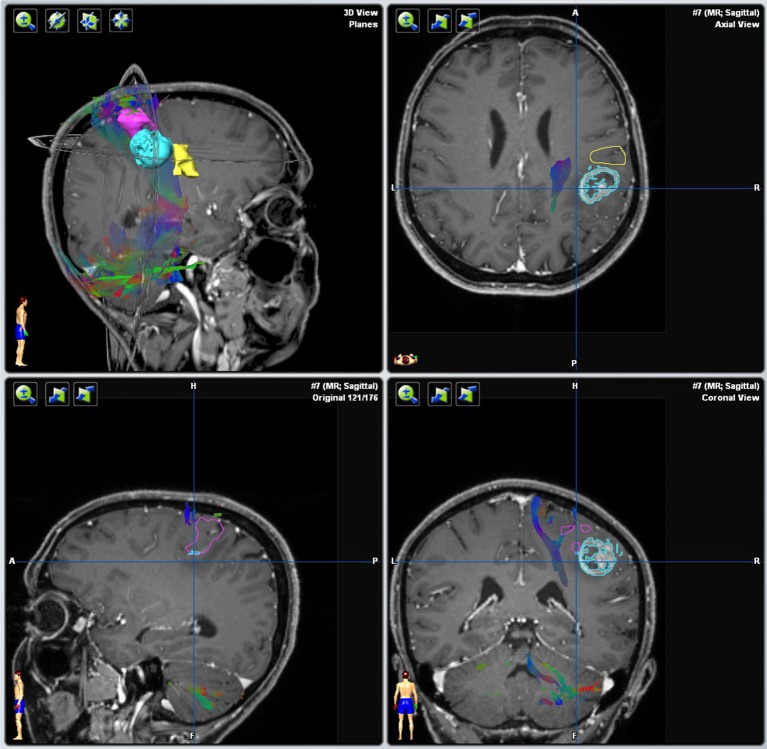
Implementation of fMRI and DTI in the neuro-navigation system [brain tumor (light blue); tumor segmentation based on the 3d cMPR-image, the hand motor association areas (magenta), the tongue motor association area (yellow), and the pyramidal tract (darker blue)].

The 65-year-old female patient had a focal seizure with paresthesia of the left upper limb and the face. Initial MRI showed a contrast-enhancing lesion in the left pre- and post-central area, distant to the midline (Figure 9). The patient additionally received motor fMRI and DTI of the pyramidal tract that was reconstructed in 3D, including the vasculature (Figure 10). Consequently, we indicated awake surgery because of the proximity to the hand and upper limb area. The patient underwent awake craniotomy under fluorescence-guidance with very intensive fluorescence staining (Figure 11). The tumor was completely removed, and no new neurological deficits developed. Early post-operative MRI confirmed gross-total resection of the lesion (Figure 12), and histology showed glioblastoma WHO IV. According to our institutional algorithm, the patient did not require a PET scan because of the intense contrast-enhancement in the initial MRI; thus, we decided on administering FL (5 mg/kg) and conducting awake surgery because navigational imaging was intraoperatively equipped with functional data (Figure 13).

#### Case #3

**Figure 14:**
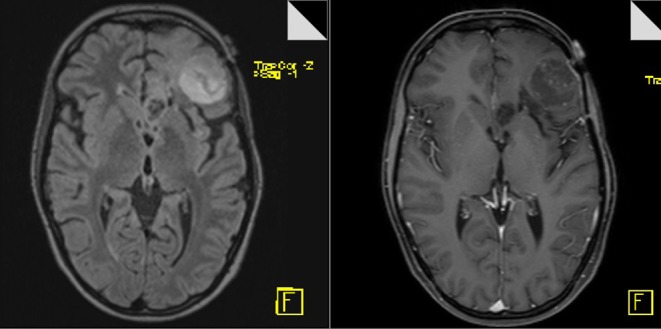
Pre-operative MR image (T1-weighted and FLAIR sequences, axial planes) showing weak contrast-enhancement and marginal edema.

**Figure 15:**
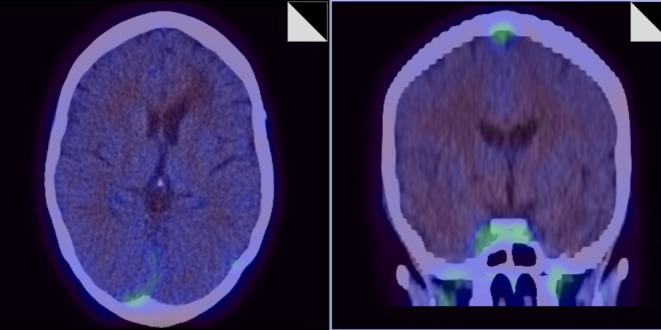
Pre-operative FET-PET showing almost absence of metabolic activity.

**Figure 16:**
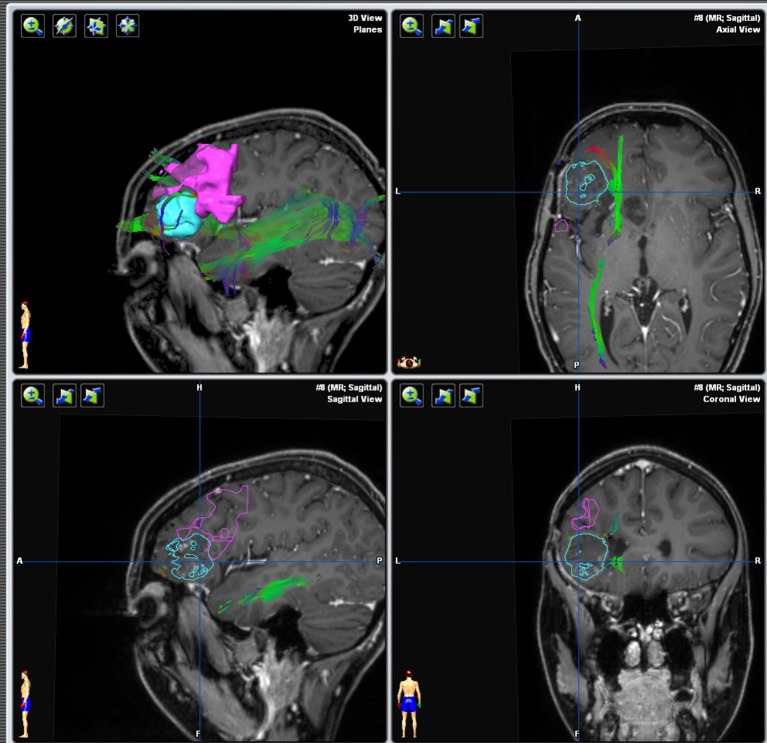
Implementation of fMRI and DTI in the neuro-navigation system [brain tumor (light blue); tumor segmentation based on a FLAIR 3dspace image (not shown), inferior frontal language association area (magenta) and ventral language pathway including uncinate fascicle, extreme capsule, inferior longitudinal fascicle and inferior front-occipital fascicle (green)].

**Figure 17:**
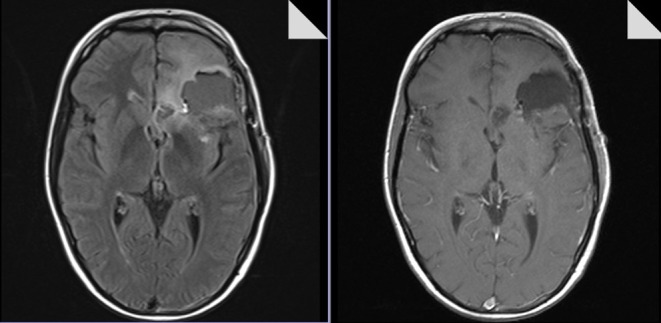
Post-operative MR image (T1-weighted and FLAIR sequences, axial planes) confirming complete removal of the tumor.

This complex case of faintly contrast-enhancing, secondary malignantly transformed oligodendroglioma WHO III, that had been initially diagnosed as astrocytoma WHO II 11 years ago, was additionally chosen to illustrate our workflow. The tumor—pre-treated with surgery, radiation, and chemotherapy—had recurred, showing marginal contrast-enhancement in the T1-weighted sequence in conventional MRI (Figure 14). Consequently, FET-PET was conducted that also showed only marginal metabolic activity (Figure 15). However, because of the recent tumor progress and the near-eloquent localization of the tumor in the left frontal cortex, we recommended fMRI followed by subsequent awake craniotomy. Intraoperatively, all imaging modalities were displayed on the navigational screen (Figure 16), and we used fluorescence-guidance with FL (5 mg/kg) and ultrasound. Weak but still usable fluorescence was detected, allowing for complete tumor removal based on the early post-operative MRI (Figure 17). The neuropathological workup showed anaplastic oligodendroglioma WHO III.

Here, all possible functional and metabolic imaging modalities were included pre- and intraoperatively. Fluorescence-guidance was helpful in this patient, although the initial MRI had only indicated moderate blood brain barrier disruption and the initial PET only demonstrated weak activity.

## Discussion

For selected patients with a brain tumor, a detailed, sophisticated workup of different neuroimaging modalities can be of the utmost importance for the pre-operative evaluation and/or simulation of the neurosurgical approach, and for choosing the technique of dissection and removal. This way, the highest possible degree of removal of neoplastic tissue can be attempted with a significant simultaneous increase in safety. Despite pre-operative metabolic and functional visualization of the targeted area by means of PET and fMRI/rsfMRI/DTI and distinctively integrated neuro-navigation, fluorescence-guided surgery with either FL, 5-ALA, or ICG plays a key role in the majority of operations. With this technique, areas of interest are visualized in real time and according to their specific properties (tumor metabolism with 5-ALA, disrupted BBB with FL, patterns of blood flow with ICG and FL). FL-based confocal endomicroscopy, which is still under scientific evaluation, can further enhance intraoperative accuracy and efficacy.

The discussed imaging modalities, however, should not be arbitrarily applied in every patient with a brain tumor, because such application would counteract the value of an individually tailored combination of these modalities. In most neurosurgical departments, this type of imaging workup is only possible with a high standard of economic and personal competence, and in the case of well-established interdisciplinary collaboration with the departments of neuroradiology, neurology, oncology, nuclear medicine, and neuropathology. Based on this cooperation, the combination of different imaging modalities carries a huge benefit for patients in terms of preserving neurological function while creating the best possible foundation for any type of adjuvant treatment (6).

However, some limitations should be clearly stated. Like any academic institution, we can only offer the technical modalities available. For example, there is no intraoperative MRI to be integrated into our IA. Furthermore, some inter-observer bias cannot be excluded, especially in the interpretation of weak or flaw fluorescence in non-contrast enhancing gliomas. Additionally, the use of fluorescence-guided confocal endomicroscopy is still under scientific evaluation.

Our IA was established 10 years ago and has been adapted according to contemporary scientific perceptions, such as fluorescence-guided surgery and the integration of FET-PET and functional data. In 2017, we published our institutional series before and after the introduction of our IA that was established simultaneously with the opening of the certified neuro-oncologic center in 2009. This analysis confirms that the implementation of the IA has not only greatly contributed to the significantly prolonged PFS and OS, but has also clearly increased the rate of gross-total resections of HGG (6). The IA has been broadly accepted across the disciplines involved in the NOC and serves as the centerpiece of our weekly neuro-oncologic tumor board.

## Author Contributions

All authors listed have made a substantial, direct and intellectual contribution to the work, and approved it for publication.

### Conflict of Interest Statement

HL was employed by the company 1000shapes GmbH, Berlin, Germany. K-MS, JH, and AB received travel funds and honoraria from Carl Zeiss Meditec, Germany. The remaining authors declare that the research was conducted in the absence of any commercial or financial relationships that could be construed as a potential conflict of interest.
